# Convergent mechanism underlying the acquisition of vertebrate scotopic vision

**DOI:** 10.1016/j.jbc.2024.107175

**Published:** 2024-03-16

**Authors:** Keiichi Kojima, Masataka Yanagawa, Yasushi Imamoto, Yumiko Yamano, Akimori Wada, Yoshinori Shichida, Takahiro Yamashita

**Affiliations:** 1 Department of Biophysics, Graduate School of Science, Kyoto University, Kyoto, Japan; 2 Faculty of Medicine, Dentistry and Pharmaceutical Sciences, Okayama University, Okayama, Japan; 3 Cellular Informatics Laboratory, RIKEN Cluster for Pioneering Research, Wako, Japan; 4 Molecular and Cellular Biochemistry, Graduate School of Pharmaceutical Sciences, Tohoku University, Sendai, Miyagi, Japan; 5 Comprehensive Education and Research Center, Kobe Pharmaceutical University, Kobe, Japan; 6 Laboratory of Organic Chemistry for Life Science, Kobe Pharmaceutical University, Kobe, Japan; 7 Research Organization for Science and Technology, Ritsumeikan University, Kusatsu, Shiga, Japan

**Keywords:** rhodopsin, visual pigment, G protein–coupled receptor (GPCR), signal transduction, molecular evolution, vision, photobiology

## Abstract

High sensitivity of scotopic vision (vision in dim light conditions) is achieved by the rods’ low background noise, which is attributed to a much lower thermal activation rate (*k*_th_) of rhodopsin compared with cone pigments. Frogs and nocturnal geckos uniquely possess atypical rods containing noncanonical cone pigments that exhibit low *k*_th_, mimicking rhodopsin. Here, we investigated the convergent mechanism underlying the low *k*_th_ of rhodopsins and noncanonical cone pigments. Our biochemical analysis revealed that the *k*_th_ of canonical cone pigments depends on their absorption maximum (λ_max_). However, rhodopsin and noncanonical cone pigments showed a substantially lower *k*_th_ than predicted from the λ_max_ dependency. Given that the λ_max_ is inversely proportional to the activation energy of the pigments in the Hinshelwood distribution-based model, our findings suggest that rhodopsin and noncanonical cone pigments have convergently acquired low frequency of spontaneous-activation attempts, including thermal fluctuations of the protein moiety, in the molecular evolutionary processes from canonical cone pigments, which contributes to highly sensitive scotopic vision.

The vertebrate retina has two types of photoreceptor cells, rods and cones, for scotopic and photopic vision, respectively (1, 2, 3). Rods and cones contain different types of visual pigments, rhodopsin and cone pigments, respectively, as photon detectors. Visual pigments commonly contain 11-*cis* retinal as a chromophore to absorb light. Upon light absorption, *cis*-*trans* photoisomerization of the retinal triggers the formation of the active state of visual pigments, which activates G protein (Gt) to induce the light-dependent hyperpolarization response of photoreceptor cells. Scotopic vision requires extremely high sensitivity and low threshold for the special ability to detect only a few photons (4, 5, 6). The electrical signals produced by single-photon absorptions in rods need to be reliably transmitted to higher-order retinal neurons even in the presence of light-independent noise, which consists of two components, discrete and continuous noise (7). The discrete noise originates from the spontaneous activation of the visual pigments (7, 8). This spontaneous formation of the active state of visual pigments occurs as a result of the thermal isomerization of the retinal chromophore in darkness. On the other hand, the continuous noise originates from the spontaneous activation of phosphodiesterase 6 in the phototransduction cascade (9, 10). The discrete noise is indistinguishable from a rod’s true single-photon response, whereas the continuous noise is eliminated as a background noise in the higher-order retinal neurons (6, 11). This means that the discrete noise level of rods in principle sets a limit for the absolute threshold in scotopic vision (6, 11, 12, 13). Therefore, the suppression of the thermal activation rate in rhodopsin enhances the sensitivity of scotopic vision. We previously elucidated how rhodopsin has evolved to acquire its low thermal activation rate. Our biochemical analysis revealed that rhodopsin achieved its low thermal activation rate by the acquisition of two amino acid residues near the retinal (*i.e.,* Glu122 and Ile189) during the molecular evolutionary process from cone pigments which exhibit much higher thermal activation rates (Fig. 1) (14).

**Figure 1:**
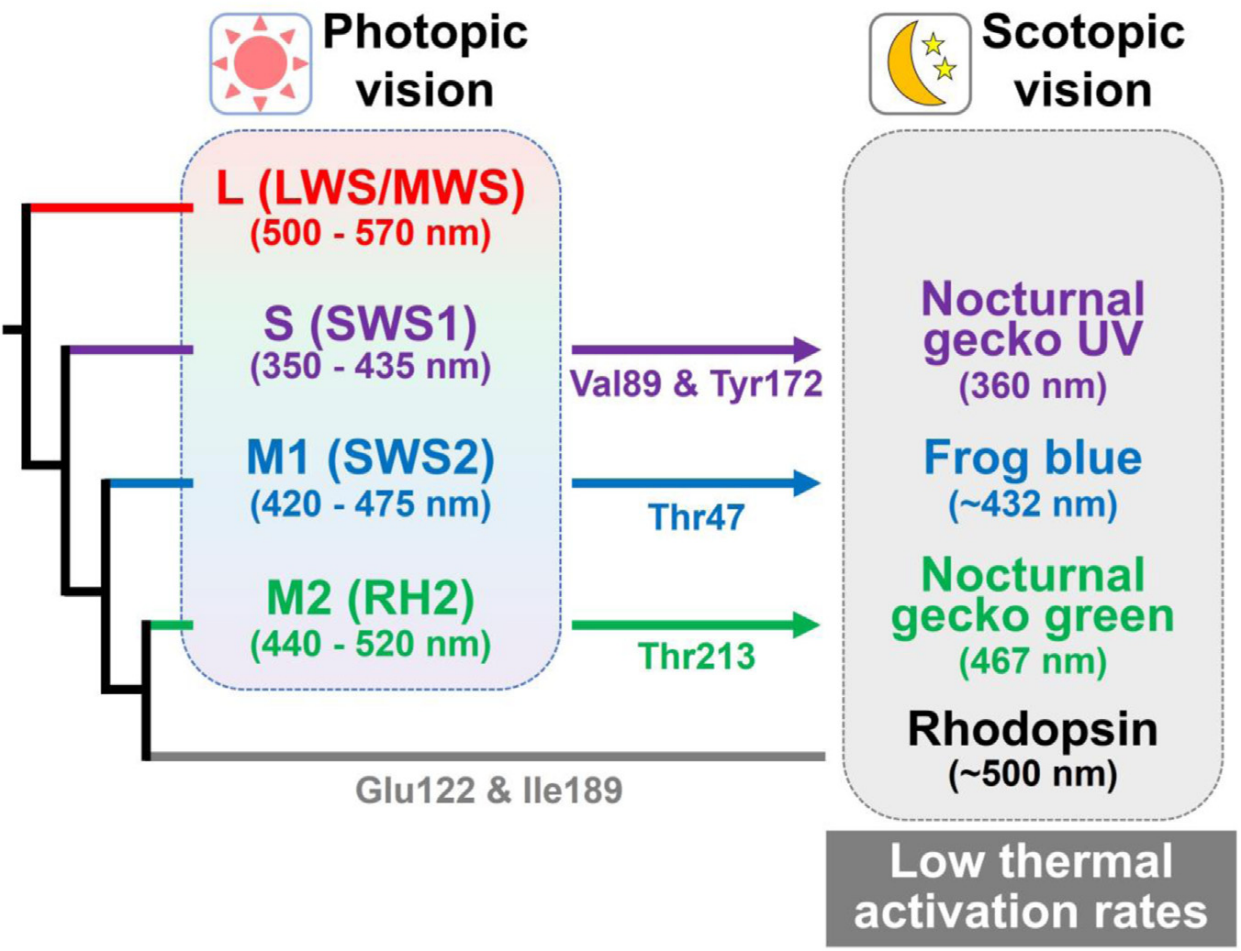
**Schematic diagram of phylogenetic relationship and absorption maxima (λ**_**max**_**) of vertebrate visual pigments.** Cone pigments are phylogenetically classified into four groups, S (SWS1), M1 (SWS2), M2 (RH2), and L (LWS/MWS) and are generally utilized for photopic vision. In the molecular evolutionary process, rhodopsin was diversified from cone pigments by the acquisition of Glu122 and Ile189 to exhibit the low thermal activation rate for scotopic vision. Frog blue cone pigments and nocturnal gecko green and UV cone pigments evolutionarily decreased the thermal activation rates by the acquisition of several amino acid residues (*i.e.*, Thr47 for frog blue, Thr213 for nocturnal gecko green, and Val89 and Tyr172 for nocturnal gecko UV) for scotopic vision. The λ_max_ values of visual pigments belonging to each group are also shown.

In addition, the retinas of several vertebrates, such as frogs (anurans) and nocturnal geckos, uniquely possess multiple types of rods for scotopic vision. This contrasts with the presence of a single type of rod containing rhodopsin and multiple types of cones containing different cone pigments in the retinas of most vertebrates. Frogs possess two types of rods, one type that contains rhodopsin and another type that contains blue-sensitive cone pigment (frog blue) (15, 16). Nocturnal geckos possess three types of rods which contain not rhodopsin but red-, green- and UV-sensitive cone pigments (nocturnal gecko red, green, and UV, respectively), and have lost the rhodopsin gene and blue-sensitive cone pigment gene from the genome (17, 18, 19, 20, 21). In these vertebrate species, the multiple types of rods having different color sensitivities are thought to be used for distinguishing colors under scotopic conditions (22, 23, 24). Our recent biochemical analysis revealed that in these noncanonical cone pigments (*i.e.,* frog blue, nocturnal gecko green, and UV) the thermal activation rates were suppressed to mimic rhodopsin, and this suppression underlies the low noise level in the atypical rods and leads to the acquisition of scotopic color vision in frogs and nocturnal geckos (25, 26). Noteworthily, we identified key amino acid residues responsible for the low thermal activation rates of noncanonical cone pigments (*i.e.*, Thr47 for frog blue, Thr213 for nocturnal gecko green, and Val89 and Tyr172 for nocturnal gecko UV) (Fig. 1).

Previous electrophysiological studies of various visual pigments assessed the regulatory mechanism of the thermal activation rates of visual pigments (8, 27, 28, 29). That is, visual pigments with longer absorption maximum (λ_max_) show higher thermal activation rates and are less suitable for scotopic vision, as originally proposed by Barlow over 60 years ago (30). Using a combination of electrophysiological and theoretical analysis, Luo *et al*. showed that the thermal activation rates (*k*_th_) of visual pigments can be quantitatively expressed based on the Hinshelwood distribution as follows:

(Eq. 1) $$k_{\text{th}}=A\times e^{-\frac{Ea}{RT}}\sum_{1}^{m}\frac{1}{\left(m-1\right)!}\;{\left(\frac{E_{a}}{RT}\right)}^{m-1}$$

where *R* is the gas constant, *T* is absolute temperature, and *m* is the number of molecular vibrational modes contributing thermal energy to pigment activation (31). According to Hinshelwood’s original theory of unimolecular reactions, *2m* can be taken as the number of degrees of freedom of the activated molecule (32). Ala-Laurila *et al*. revisited Hinshelwood’s theory to explain the discrepancy between the photo and thermal activation energy of rhodopsin (27). When only the retinal degrees of freedom are considered, *m* has a maximum value of 141, but it can be even larger when the entire rhodopsin is considered. Here, the activation energy *E*_a_ is inversely proportional to the pigment’s λ_max_ (*E*_a_ = 0.84 × *h*c/λ_max_) and the preexponential factor *A* is the frequency of retinal thermal isomerization attempts derived from the protein molecule and primarily represents the structural fluctuation of the retinal binding pocket (RBP) (31, 33). Thus, the thermal activation rates of visual pigments are determined by two factors: the pigment’s λ_max_ and the preexponential factor *A* (31, 33). This suggests the possibility that visual pigments have two potential strategies, the blue-shift of the pigment’s λ_max_ and the decrease in the preexponential factor *A*, to achieve the low thermal activation rates. According to this framework, visual pigments with shorter λ_max_ (*e.g.*, UV-sensitive pigments) tend to exhibit lower thermal activation rates and are more suitable for scotopic vision than those with longer λ_max_ (*e.g.*, red-sensitive pigments). The phylogenetic relationship of the visual pigments suggests that noncanonical cone pigments of frogs and nocturnal geckos convergently acquired their low thermal activation rates to mimic that of rhodopsin (Fig. 1). This raises the question of whether the visual pigments working in rods, namely rhodopsin and noncanonical cone pigments, used the same or distinct strategies to decrease the thermal activation rates despite their different spectral sensitivities. In this study, to understand the convergent mechanism underlying the acquisition of scotopic vision, we elucidated how rhodopsin and noncanonical cone pigments achieved the low thermal activation rates based on a biochemical analysis technique that we developed. First, we comprehensively analyzed the thermal activation rates of canonical and noncanonical cone pigments which show different λ_max_ ranging from the UV to the visible region (360–510 nm). We verified that the thermal activation rates of canonical cone pigments that we measured can be expressed by the Hinshelwood distribution-based model (Equation 1) assuming *E*_a_ = 0.84 × *h*c/λ_max_ as reported in the previous electrophysiological analysis (31). According to this relationship, we quantitatively determined the contribution of the pigment’s λ_max_ and the preexponential factor *A* to the low thermal activation rates of rhodopsin and noncanonical cone pigments. Based on these results, we discuss the mechanistic convergence in which the visual pigments working in rods suppressed their thermal activation rates by the regulation of their λ_max_ and preexponential factor *A* for scotopic vision.

## Results

### Quantitative relationship between thermal activation rates and λ_max_ in canonical cone pigments

We previously developed a biochemical technique to evaluate the thermal activation rates of recombinant visual pigments purified from cultured cells as an alternative to the electrophysiological techniques using intact photoreceptor cells (14, 25, 26, 34). This method enables us to quantitatively compare the thermal activation rates of visual pigments under the same experimental conditions. We can calculate the thermal activation rates (*k*_th_) of visual pigments using three experimentally determined values as follows (Fig. S1). First, we assumed a two-step reaction of thermal activation and deactivation of visual pigments, as shown in Fig. S1, where R and R∗ indicate visual pigments in the dark (inactive) and active states, respectively. Given that the thermal activation of R occurs much more slowly than the decay of R∗ (*k*_th_ << *k*_d_), a steady-state approximation can be applied to the concentration of R∗ and the following equation is obtained (here, *k*_d_ is the spontaneous decay rate of the activated pigment):

(Eq. 2) $$\left[R^{*}\right]=\frac{k_{\mathrm{th}}}{k_{d}}{\left[R\right]}_{0}$$

where [R]_0_ is the initial concentration of the visual pigment. In this condition, it can be considered that there is a low but constant concentration of R∗ in a solution of the purified pigments in the dark. Since R∗ is essentially the same as the light-induced Meta II intermediate, the initial rate of G protein activation by a pigment in the dark (*v*_dark_) can be approximated by the following equation:

(Eq. 3) $$v_{\text{dark}}=\frac{k_{\text{th}}}{k_{d}}v_{\text{light}}$$

where *v*_light_ is the initial rate of G protein activation by a photoactivated pigment. Therefore, we can estimate *k*_th_ from three experimentally determined values (*v*_dark_, *v*_light_, and *k*_d_) as follows:

(Eq. 4) $$k_{\text{th}}=\frac{v_{\text{dark}}}{v_{\text{light}}}k_{d}$$

**Figure 2:**
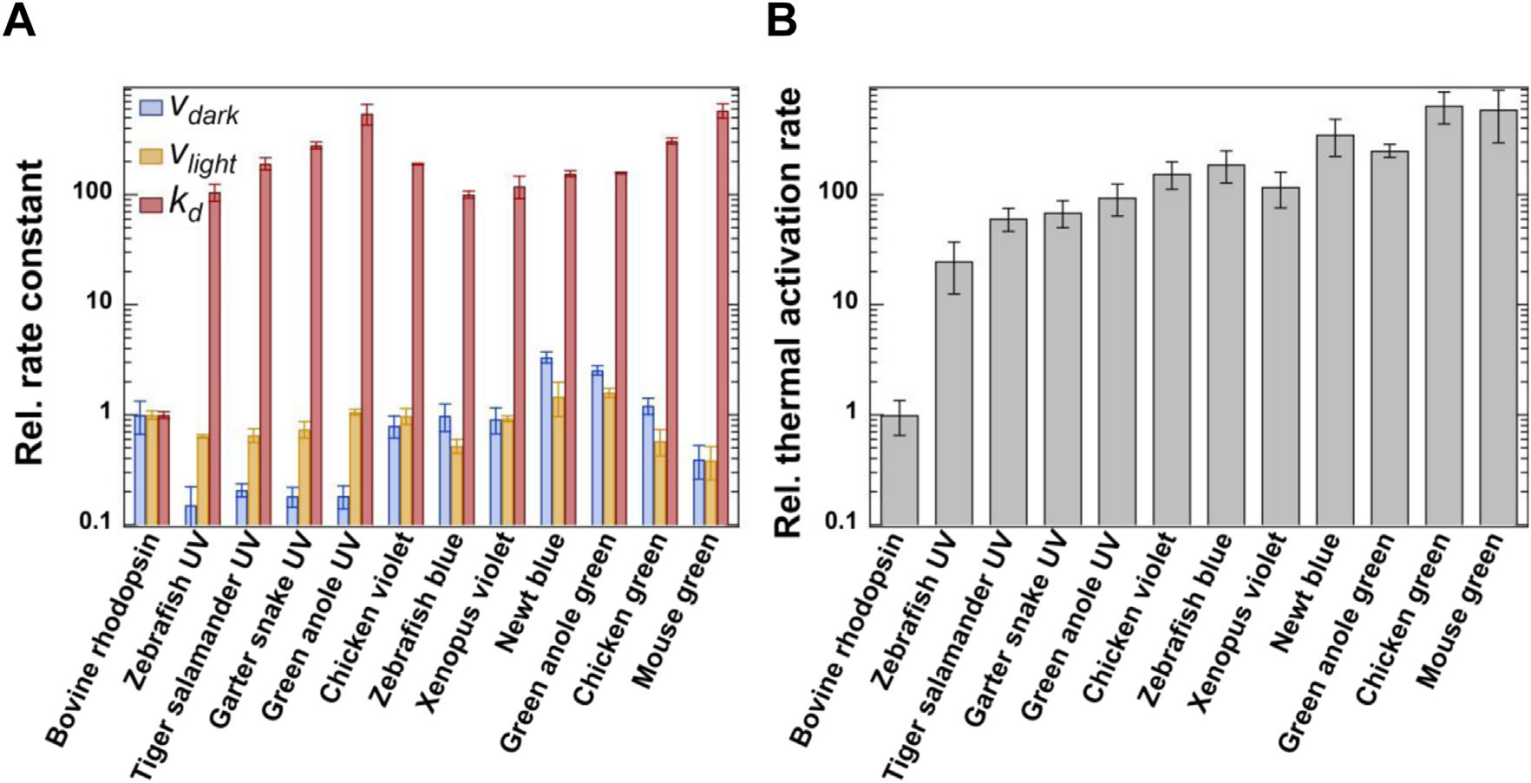
**Thermal activation rates (*k***_**th**_**) of canonical cone pigments.***A*, comparison of *v*_dark_, *v*_light,_ and *k*_d_ of bovine rhodopsin, zebrafish UV, tiger salamander UV, garter snake UV, green anole UV, chicken violet, zebrafish blue, *Xenopus* violet, newt blue, green anole green, chicken green, and mouse green measured by the biochemical and fluorescence assays (Figs. S2–S4). The values for tiger salamander UV, garter snake UV, green anole UV, zebrafish blue, newt blue, green anole green, chicken green, and mouse green were referred to in our previous studies (14, 25, 26). *n* = 3 (zebrafish UV, tiger salamander UV, garter snake UV and green anole green), 4 (green anole UV, chicken violet, zebrafish blue, *Xenopus* violet, newt blue, chicken green and mouse green), and 5 (bovine rhodopsin) for *v*_dark_; n = 3 (bovine rhodopsin, zebrafish UV, tiger salamander UV, garter snake UV, green anole UV, chicken violet, zebrafish blue, *Xenopus* violet, newt blue, green anole green, and chicken green), and 6 (mouse green) for *v*_light_; n = 3 (bovine rhodopsin, zebrafish UV, tiger salamander UV, garter snake UV, green anole UV, zebrafish blue, newt blue, and green anole green), 4 (chicken violet and *Xenopus* violet), 5 (mouse green), and 8 (chicken green) for *k*_d_. *B*, comparison of the thermal activation rates (*k*_th_) of bovine rhodopsin, zebrafish UV, tiger salamander UV, garter snake UV, green anole UV, chicken violet, zebrafish blue, *Xenopus* violet, newt blue, green anole green, chicken green, and mouse green. The *k*_th_ of bovine rhodopsin, zebrafish UV, chicken violet and *Xenopus* violet were estimated from the data presented in panel A. The *k*_th_ of tiger salamander UV, garter snake UV, green anole UV, zebrafish blue, newt blue, green anole green, chicken green and mouse green were referred to in our previous studies (14, 25, 26). All error bars represent the S.D.

Cone pigments are phylogenetically classified into four groups: S (SWS1), M1 (SWS2), M2 (RH2), and L (LWS/MWS) (35, 36). This classification corresponds well to the pigment’s λ_max_, which ranges from the UV to the visible region (approx. 350–570 nm) (Fig. 1). First, to analyze the relationship between the thermal activation rates and the pigment’s λ_max_, we compared the thermal activation rates of canonical cone pigments, which are expressed in the cones, among the four groups. We measured the thermal activation rates of the following canonical cone pigments: mouse green-sensitive cone pigment (mouse green, λ_max_ = 508 nm) from the L (LWS/MWS) group, chicken green-sensitive cone pigment (chicken green, λ_max_ = 507 nm) and green anole green-sensitive cone pigment (green anole green, λ_max_ = 500 nm) from the M2 (RH2) group, and zebrafish blue-sensitive cone pigment (zebrafish blue, λ_max_ = 418 nm) and newt blue-sensitive cone pigments (newt blue, λ_max_ = 474 nm) from the M1 (SWS2) group (Figure 1, Figure 2 and Table 1) (14, 25, 26). In the M1 (SWS2) group, the thermal activation rate of zebrafish blue is more than 2-fold lower than that of newt blue. In addition, the rate of newt blue is lower than those of green anole green, chicken green, and mouse green (Fig. 2 and Table 1). Considering the pigment’s λ_max_, this observation is consistent with the principle that visual pigments with longer λ_max_ show higher thermal activation rates.

**Table 1:** Thermal activation rate constants and absorption maxima (λ_max_) of visual pigments

Group	Visual pigment	λ_max_ (nm)	*f*_*≥E*a_^a^	*A*^b^	*k*_th_ (1/sec)^c^
Rhodopsin (RH1)	Bovine rhodopsin	500	9.85 × 10^−1^	2.51 × 10^−8^	2.47 × 10^−8^
	Mouse rhodopsin	500	9.85 × 10^−1^	2.08 × 10^−8^	2.05 × 10^−8^
	*Xenopus* rhodopsin	500	9.85 × 10^−1^	1.61 × 10^−8^	1.59 × 10^−8^
L (LWS/MWS)	Mouse green	508	9.90 × 10^−1^	1.48 × 10^−5^	1.46 × 10^−5^
M2 (RH2)	Chicken green	507	9.89 × 10^−1^	1.62 × 10^−5^	1.60 × 10^−5^
	Green anole green	500	9.85 × 10^−1^	6.32 × 10^−6^	6.22 × 10^−6^
M1 (SWS2)	Newt blue	474	9.49 × 10^−1^	9.18 × 10^−6^	8.71 × 10^−6^
	Zebrafish blue	418	6.59 × 10^−1^	7.08 × 10^−6^	4.67 × 10^−6^
S (SWS1)	*Xenopus* violet	428	7.40 × 10^−1^	3.93 × 10^−6^	2.91 × 10^−6^
	Chicken violet	416	6.40 × 10^−1^	5.97 × 10^−6^	3.82 × 10^−6^
	Green anole UV	360	1.29 × 10^−1^	1.80 × 10^−5^	2.33 × 10^−6^
	Garter snake UV	360	1.29 × 10^−1^	1.32 × 10^−5^	1.70 × 10^−6^
	Tiger salamander UV	357	1.12 × 10^−1^	1.34 × 10^−5^	1.50 × 10^−6^
	Zebrafish UV	356	1.06 × 10^−1^	5.78 × 10^−6^	6.15 × 10^−7^
M1 (SWS2)	*Xenopus* blue	432	7.68 × 10^−1^	6.74 × 10^−8^	5.17 × 10^−8^
	American bullfrog blue	431	7.58 × 10^−1^	1.38 × 10^−7^	1.05 × 10^−7^
	Mantelline frog blue	432	7.68 × 10^−1^	8.83 × 10^−8^	6.78 × 10^−8^
M2 (RH2)	Tokay gecko green	467	9.32 × 10^−1^	2.68 × 10^−7^	2.50 × 10^−7^
S (SWS1)	Tokay gecko UV	360	1.29 × 10^−1^	1.28 × 10^−6^	1.65 × 10^−7^

a The *f*_≥*E*__a_ values were calculated using Equation 5.

b The *A* values were calculated based on their thermal activation rates and *f*_*≥Ea*_ values.

c The thermal activation rates (*k*_th_) of visual pigments are the experimental data estimated using the biochemical methods from this study and our previous studies (14, 25, 26). It is noted that the absolute values of the *k*_th_ estimated by our biochemical method (*e.g.*, 2.05 × 10^−8^ s^−1^ for mouse rhodopsin) are ∼300-fold higher than those estimated by the electrophysiological method (6.64 × 10^−11^ s^−1^ for mouse rhodopsin) (40). This difference is mainly explained by the underestimation of *v*_light_ in our biochemical measurement, as discussed in the previous report (26).

Next, we focused on the S (SWS1) group, whose members are the shortest wavelength-sensitive cone pigments (Fig. 1). Cone pigments in the S (SWS1) group are divided into two types, violet- and UV-sensitive ones, based on their λ_max_ (around 400–440 nm and 360 nm, respectively). The λ_max_ of violet-sensitive pigments in the S (SWS1) group, such as chicken violet-sensitive cone pigment (chicken violet, λ_max_ = 416 nm) and *Xenopus* violet-sensitive cone pigment (*Xenopus* violet, λ_max_ = 428 nm), are equivalent to that of zebrafish blue (λ_max_ = 418 nm) in the M1 (SWS2) group (Fig. S5 and Table 1). By contrast, the λ_max_ of UV-sensitive pigments in the S (SWS1) group, such as zebrafish UV-sensitive cone pigment (zebrafish UV, λ_max_ = 356 nm), tiger salamander UV-sensitive cone pigment (tiger salamander UV, λ_max_ = 357 nm), green anole UV-sensitive cone pigment (green anole UV, λ_max_ = 360 nm), and garter snake UV-sensitive cone pigments (garter snake UV, λ_max_ = 360 nm), are >40 nm blue-shifted from those of violet-sensitive pigments (Fig. S5 and Table 1). Our analysis showed that the thermal activation rates of chicken violet and *Xenopus* violet are about 160- and 120-fold higher than that of bovine rhodopsin, respectively, and are comparable to that of zebrafish blue in the M1 (SWS2) group (Fig. 2 and Table 1). In addition, we analyzed the thermal activation rates of zebrafish UV, tiger salamander UV, green anole UV, and garter snake UV (Fig. 2 and Table 1). We estimated the thermal activation rate of zebrafish UV to be about 25-fold higher than that of bovine rhodopsin and comparable to those of tiger salamander UV, green anole UV, and garter snake UV, which we reported previously (Fig. 2 and Table 1). Thus, in general, the rates of UV-sensitive cone pigments are several-fold lower than those of violet-sensitive cone pigments in the S (SWS1) group. This is also consistent with the relationship where the blue-shift of the λ_max_ lowers the thermal activation rate of visual pigments.

**Figure 3:**
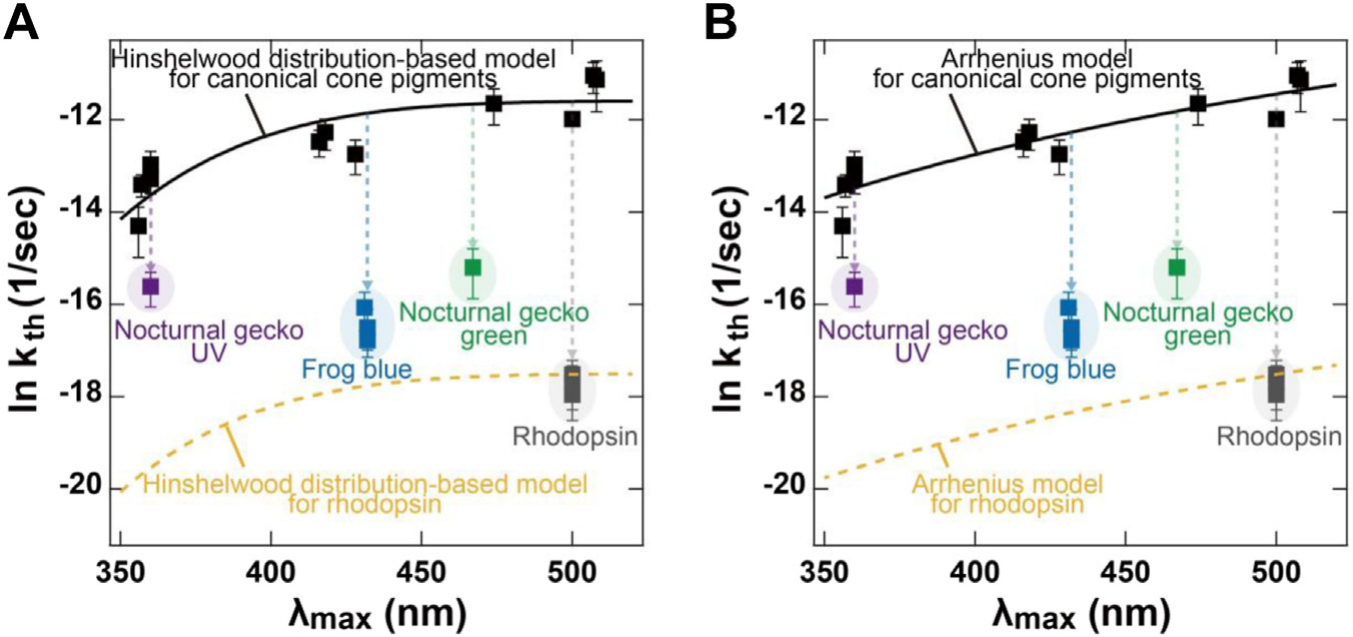
**Relationship of thermal activation rates (*k***_**th**_**) and absorption maxima (λ**_**max**_**) of visual pigments.** The relationship of thermal activation rates and λ_max_ of visual pigments was analyzed by the Hinshelwood distribution-based model (*A*) and the Arrhenius model (*B*). *Black squares* show the data of canonical cone pigments (*i.e.*, mouse green, chicken green, green anole green, newt blue, zebrafish blue, chicken violet, *Xenopus* violet, green anole UV, garter snake UV, tiger salamander UV and zebrafish UV). The plots are well fitted by the Hinshelwood distribution-based model (Equation 1) at 20 °C with *E*_a_ = 0.84 × *h*c/λ_max_, *m* = 103 and *A* = 9.23 × 10^−6^ s^−1^ (*black solid line in panel A*), and the Arrhenius model (Equation 6) at 20 °C with *E*_a_ = 1526/λ_max_ + 18.95 kcal/mol (*E*_a_ = 22 kcal/mol at 500 nm) and *A* = 2.76 × 10^11^ sec^−1^ (*black solid line in panel B*). The *k*_th_ of rhodopsins (*i.e.*, bovine, mouse, and *Xenopus* rhodopsin), frog blue (*i.e. Xenopus*, American bullfrog, and mantelline frog blue), nocturnal gecko green and UV (*i.e.*, tokay gecko green and tokay gecko UV, respectively) are also shown as *gray*, *blue*, *green*, and *violet squares*, respectively (14, 25, 26). The *yellow dashed lines* represent the Hinshelwood distribution-based model (Equation 1) for rhodopsin at 20 °C with *E*_a_ = 0.84 × *h*c/λ_max_, *m* = 103 and *A* = 2.51 × 10^–8^ s^–1^ in *panel A*, and the Arrhenius model (Equation 6) for rhodopsin at 20 °C with *E*_a_ = 1526/λ_max_ + 18.95 kcal/mol (*E*_a_ = 22 kcal/mol at 500 nm) and *A* = 6.40 × 10^8^ sec^–1^ in *panel B*. All error bars represent the S.D. All of the data are listed in Table 1.

To quantitatively analyze the relationship between the thermal activation rates and λ_max_ among canonical cone pigments working in the cones, we plotted their rates against λ_max_ (black squares in Fig. 3*A*) (14, 25). The plots clearly suggest a relationship whereby cone pigments with longer λ_max_ show higher thermal activation rates. According to a previous study (31), we sought to fit the plots of canonical cone pigments with the Hinshelwood distribution-based model (Equation 1), assuming *E*_a_ = 0.84 × *h*c/λ_max_. It should be noted that a fitting analysis was performed to determine the best-fit curve of the datasets with the lowest sum of squares when both *m* and *A* are variable (1 ≤ *m* ≤ 141) (Fig. S6). The plots of the cone pigments were fitted well by the model with *m* = 103 and *A* = 9.23 × 10^−6^ s^−1^, with an Akaike information criterion (AIC) value of 22.9 (black solid line in Fig. 3*A*). Thus, we successfully verified that the thermal activation rates of canonical cone pigments estimated by the biochemical analysis can be expressed by the Hinshelwood distribution-based model (Equation 1). We also defined a pigment molecule’s probability (*f*_*≥E*a_) of having relevant thermal energy ≥*E*_a_, which represents the effects of the pigment’s λ_max_ on the Hinshelwood distribution-based model as follows:

(Eq. 5) $$f_{\geq Ea}=e^{-\frac{Ea}{RT}}\sum_{1}^{m}\frac{1}{\left(m-1\right)!}\;{\left(\frac{E_{a}}{RT}\right)}^{m-1}$$

Using Equation 5 with *E*_a_ = 0.84 × *h*c/λ_max_ and *m* = 103, the effects of the pigment’s λ_max_ (*i.e.*, *f*_*≥E*a_) on the thermal activation rates can be evaluated (Table 1).

### Contribution of the λ_max_ and preexponential factor *A* to the low thermal activation rates of rhodopsin and noncanonical cone pigments

In the previous studies, we estimated the thermal activation rates of not only rhodopsin but also noncanonical cone pigments expressed in the rods of frogs and nocturnal geckos (14, 25, 26). To investigate the contribution of the λ_max_ and the preexponential factor *A* to the low thermal activation rates of visual pigments working in the rods in the Hinshelwood distribution-based model, we plotted the rates of rhodopsins (bovine, mouse and *Xenopus* rhodopsin) and of noncanonical cone pigments, namely, *Xenopus*, American bullfrog and mantelline frog blue-sensitive cone pigments (*Xenopus*, American bullfrog, and mantelline frog blue, respectively) and tokay gecko green- and UV-sensitive cone pigments (tokay gecko green and UV), in Figure 3*A*. The rates estimated for rhodopsin and noncanonical cone pigments are downshifted from the curve of the Hinshelwood distribution-based model fitted for the rates estimated for canonical cone pigments (black solid line in Fig. 3*A*). This implies that the pre-exponential factor *A* values of rhodopsin and noncanonical cone pigments are lower than those of canonical cone pigments. We calculated the *f*_*≥E*a_ values of rhodopsin and noncanonical cone pigments using Equation 5 (Table 1). Subsequently, we evaluated their *A* values using the thermal activation rates and *f*_*≥E*a_ values (Table 1). The *A* values of bovine, mouse, and *Xenopus* rhodopsin are substantially lower by 370-, 440-, and 570-fold, respectively, than those of canonical cone pigments which were determined by fitting all data of their thermal activation rates (*i.e.*, 9.23 × 10^−6^ s^−1^). Similarly, the *A* values of noncanonical cone pigments are also lower, but to a lesser extent. That is, *Xenopus* blue, tokay gecko green, and tokay gecko UV exhibit 140-, 34-, and 7-fold lower *A* values than canonical cone pigments, respectively. Thus, rhodopsin and noncanonical cone pigments decreased the preexponential factor *A*, although to different levels, which resulted in the convergent acquisition of the low thermal activation rates irrespective of the pigment’s λ_max_. However, the detailed comparison shows that the difference in the thermal activation rates among the visual pigments in the rods cannot be fully explained by the difference in their *A* values. The *A* value of tokay gecko UV is 5-fold higher than that of tokay gecko green, whereas the thermal activation rates of tokay gecko green and UV are comparable to each other. Considering the 7-fold lower *f*_*≥E*a_ value of tokay gecko UV compared to that of tokay gecko green, the spectral blue-shift in tokay gecko UV can compensate for the small suppression level of the *A* value to achieve the low thermal activation rate equivalent to that of tokay gecko green. Hence, the low thermal activation rate of nocturnal gecko UV can be attributed to the simultaneous suppression of the *A* and *f*_*≥E*a_ values. This contrasts with the findings that the low rates of rhodopsin, frog blue and nocturnal gecko green, can primarily be ascribed to the suppression of the *A* value alone. Remarkably, among the visual pigments utilized for scotopic vision, rhodopsins exhibit the lowest *A* values to acquire the lowest thermal activation rate (Table 1). In Figure 3*A*, we downshifted the fitting curve for canonical cone pigments (black solid line) to pass the *A* value of bovine rhodopsin (*i.e.*, 2.51 × 10^−8^ s^−1^) (yellow dashed line). This highlights the finding that the curve of the Hinshelwood distribution-based model for rhodopsin is lower than the thermal activation rates of noncanonical cone pigments (yellow dashed line in Fig. 3*A*). This means that rhodopsin decreased the *A* value, probably by suppression of the structural fluctuation, compared to cone pigments, which led to the lowest thermal activation rate among visual pigments analyzed so far in our study.

### Arrhenius model for the relationship between thermal activation rates and λ_max_ of visual pigments

In contrast to the Hinshelwood distribution-based model (Equation 1), previous studies utilized the basic Arrhenius model for the thermal activation rates of toad rhodopsin to estimate the activation energy as 22 kcal/mol (7, 37). Then, we reexamined whether the thermal isomerization rates can be expressed based on the Arrhenius model, in which the Boltzmann distribution is implicit, as follows:

(Eq. 6) $$k_{\mathrm{th}}=A\times \exp\;\left(-\frac{E_{a}}{RT}\right)$$

We assumed that *E*_a_ is proportional to the reciprocal of the pigment’s λ_max_ (*E*_a_ = a/λ_max_ + b kcal/mol) and is estimated to be 22 kcal/mol at 500 nm based on a previous electrophysiological study (7). Fitting analysis was performed to determine the best-fit curve of the datasets with the lowest sum of squares, with both a and b as variables and a/500 + b fixed at 22 kcal/mol. The plots of canonical cone pigments are well fitted by the Arrhenius model with *A* = 2.76 × 10^11^ sec^−1^ and *E*_a_ = 1526/λ_max_ + 18.95 kcal/mol (black solid line in Fig. 3*B*), like the fitting curve of the Hinshelwood distribution-based model (black solid line in Fig. 3*A*). The AIC value of the Arrhenius model was estimated to be 17.6, which is close to the value of the Hinshelwood distribution-based model (*i.e.*, 22.9). This implies that the Arrhenius model can explain the relationship between the thermal activation rates and λ_max_, similar to the Hinshelwood distribution-based model (Fig. 3*A*). In this analysis, the rates of rhodopsin and noncanonical cone pigments are downshifted from the curve of the Arrhenius model fitted for the rates of canonical cone pigments (black solid line in Fig. 3*B*). We calculated the values of *A* and exp (-*E*_a_/RT) in the Arrhenius model (Table S1). As in the case of the analysis by the Hinshelwood distribution-based model, the *A* values of rhodopsin and noncanonical cone pigments are substantially lower than those of canonical cone pigments (*i.e.*, 2.76 × 10^11^ s^−1^), although to different levels. Furthermore, we downshifted the fitting curve to pass the *A* value of bovine rhodopsin and confirmed that the curve for rhodopsin is lower than the thermal activation rates of noncanonical cone pigments (yellow dashed line in Fig. 3*B*). This means that rhodopsin shows the lowest thermal activation rate among visual pigments irrespective of the pigment’s λ_max_. These observations in the Arrhenius model are consistent with those in the Hinshelwood distribution-based model.

## Discussion

Comparison of the thermal activation rates of visual pigments measured by electrophysiological studies using intact photoreceptor cells (7, 27, 28, 33, 37, 38, 39, 40) has provided valuable information about the regulatory models for the relationship of thermal activation rates and λ_max_ of visual pigments. Baylor *et al*. determined the Arrhenius activation energy (*E*_a_ = 22 kcal/mol) from the temperature dependence of the thermal activation rates of toad rhodopsin. The measured activation energy of the thermal activation was lower than the observed energy storage (∼32 kcal/mol) in the batho intermediate, an early photointermediate after the photoisomerization of the retinal, of bovine rhodopsin. This discrepancy in the energy gap caused controversy regarding whether the formation of the active state (Meta II intermediate) in the thermal activation process occurs by a pathway different from the photoactivation pathway through the batho intermediate. To address this issue, Ala-Laurila *et al*. proposed the application of the Hinshelwood distribution instead of the Boltzmann distribution to estimate the activation energy, assuming that the internal energy present in the many vibrational modes of the chromophore is taken into account (27). Luo *et al*. further developed this theory into the Hinshelwood distribution-based model (Equation 1), where the activation energy *E*_a_ is inversely proportional to the pigment’s λ_max_ (*E*_a_ = 0.84 × *h*c/λ_max_) (31). These analyses explained the electrophysiological data in line with Barlow's hypothesis (30). In this scheme, the activation energy for rhodopsin was estimated to be ∼48 kcal/mol, which is higher that the energy for the batho intermediate. Importantly, the thermal activation rates of canonical cone pigments estimated by our biochemical analysis can be expressed by the Hinshelwood distribution-based model (Fig. 3*A*), which supports the notion that thermal and photo activation follow the same molecular pathway. On the other hand, the rates of canonical cone pigments were also well fitted by the simpler Arrhenius model with *E*_a_ = 22 kcal/mol at 500 nm (Fig. 3*B*), which assumes that the thermal activation follows a pathway different from the photoactivation pathway and bypasses the formation of the batho intermediate. Although the Hinshelwood distribution-based model (Equation 1) has commonly been utilized as a primary equation to explain the thermal activation rates (31, 33), this study could not determine which model was more plausible. The validity of the two models (the Hinshelwood distribution-based model *versus* the Arrhenius model) will be investigated by future computational analysis of the dependency of the activation energy (*E*_*a*_) on the λ_max_ in canonical cone pigments.

**Figure 4:**
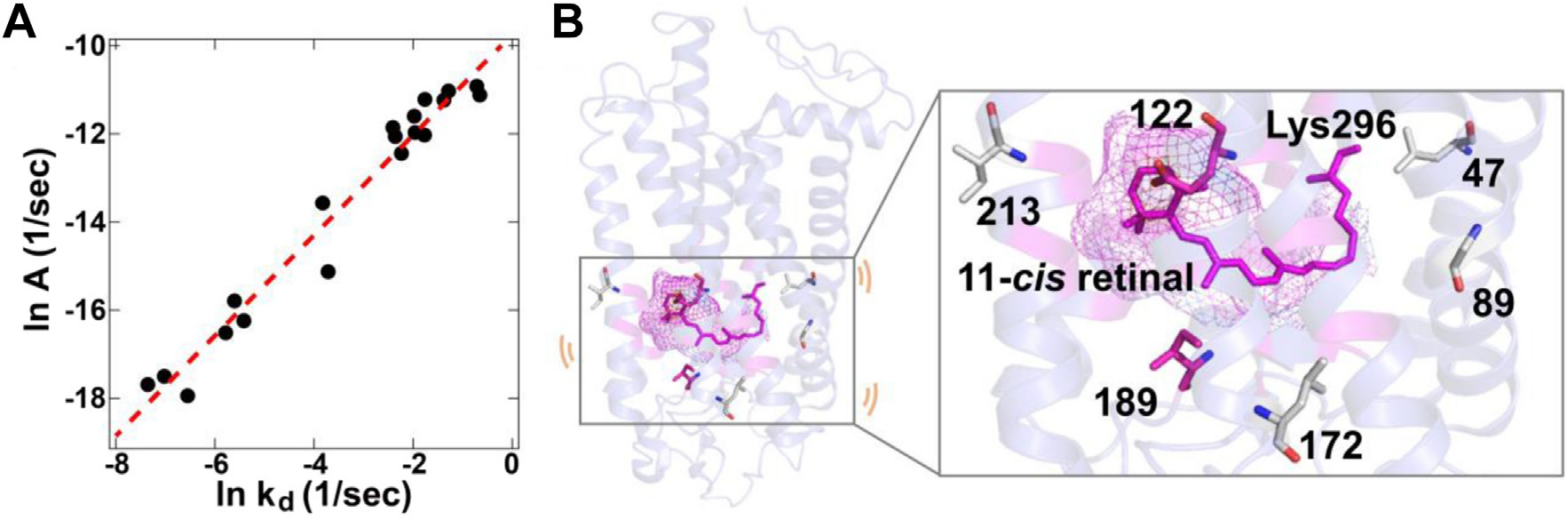
**Regulatory mechanism of the thermal activation of visual pigments.***A*, correlation between the preexponential factor *A* and *k*_d_ of the visual pigments. Plots of ln *A* and ln *k*_d_ of canonical cone pigments (*i.e.*, mouse green, chicken green, green anole green, newt blue, zebrafish blue, chicken violet, *Xenopus* violet, green anole UV, garter snake UV, tiger salamander UV, and zebrafish UV), rhodopsins (*i.e.*, bovine, mouse, and *Xenopus* rhodopsin), frog blue (*i.e. Xenopus*, American bullfrog and mantelline frog blue), nocturnal gecko green and UV (*i.e.*, tokay gecko green and tokay gecko UV, respectively). The regression line derived from all data is shown by a *red dashed line*. *R* = 0.98 (*p* < 0.01). *B*, key amino acid residues for the low thermal activation rates in rhodopsins and noncanonical cone pigments. These residues possibly suppress the structural fluctuation of the RBP in the dark state. This figure was constructed based on the crystal structures of the dark state of bovine rhodopsin (1U19 (49)). The cavities around the RBP are shown in the *magenta* mesh. Lys296 and 11-*cis* retinal are also denoted as *sticks*. RBP, retinal binding pocket.

A previous study argued that the preexponential factor *A* value primarily represents the structural fluctuation of the RBP (open *versus* closed) in the Hinshelwood distribution-based model (Equation 1) (33). By applying the Eyring equation, the preexponential factor *A* is accounted for by the value of ΔS^‡^, which reflects the fluctuation of a visual pigment. Notably, our results showed that there is a strong correlation between the *A* value and the *k*_d_ in visual pigments (Fig. 4*A*). Considering that the *k*_d_ is affected by the structural fluctuation of the RBP, this correlation also supports the concept that the *A* value can be regulated by the structural fluctuation of the RBP. Our previous mutational analysis revealed that Glu122 and Ile189, which are conserved among most rhodopsins and located on the surface of the RBP, function as key residues responsible for the low thermal activation rate of rhodopsin (Figs. 4*B* and S7) (14). The two residues of rhodopsin are well adjusted to suppress the fluctuation of the RBP by the arrangement of the hydrogen-bonding network surrounding the RBP through the direct interaction with the retinal. Our additional mutational analysis identified the key residues responsible for the low thermal activation rate of noncanonical cone pigments (*i.e.*, Thr47 for frog blue, Thr213 for nocturnal gecko green, and Val89 and Tyr172 for nocturnal gecko UV) (25, 26). The substitutions of these residues substantially increased the thermal activation rates, which shows that these residues suppress the thermal activation rates in noncanonical cone pigments. This indicates that the acquisition of the low thermal activation rates in rhodopsin and noncanonical cone pigments was not due to sequence convergence. In contrast to Glu122 and Ile189, the key residues of noncanonical cone pigments are thought to be located on the outside of the RBP (Figs. 4*B* and S7). These residues of noncanonical cone pigments possibly suppress the fluctuation of the RBP through the interaction with the surrounding residues consisting of the RBP. This difference supports an evolutionary scenario in which rhodopsin and noncanonical cone pigments were optimized for scotopic vision by mechanistic convergence based on distinct sequence changes (41).

In the Hinshelwood distribution-based model (Equation 1), the activation energy *E*_a_ is inversely proportional to the pigment’s λ_max_ (*E*_a_ = 0.84 × *h*c/λ_max_) (31). Gozem *et al*. proposed that the retinal of rhodopsin exhibits a transition state with charge-transfer (TS_CT_) in the thermal activation path, which has the same electronic structure as the excited-state in the canonical path of photoisomerization of 11-*cis* retinal in rhodopsin by computational calculation methods (42). This calculation predicted that *E*_a_ is proportional to the reciprocal of λ_max_, which rationally explains the regulatory model of the thermal activation reported in Luo *et al*. (31). However, Gozem *et al*. also predicted that the retinal of rhodopsin has another transition state with diradical character (TS_DIR_). The TS_DIR_ rather than the TS_CT_ controls the thermal isomerization of retinal in rhodopsin whose λ_max_ is below 470 nm and has a lower energy barrier as λ_max_ of rhodopsin shortens. This prediction implies that visual pigments with a λ_max_ below 470 nm show higher thermal activation rates when their λ_max_ values are shorter, which contradicts the relationship in which visual pigments with longer λ_max_ show higher thermal activation rates (42). Our results revealed that the rates of canonical cone pigments whose λ_max_ range from the UV to the visible region (360–510 nm) are expressed by the Hinshelwood distribution-based model assuming *E*_a_ = 0.84 × *h*c/λ_max_. This observation clarified the principle that visual pigments with longer λ_max_ show higher thermal activation rates even in the region below 470 nm, contrary to the prediction of Gozem *et al*. (42). Considering that Gozem *et al*. calculated the isomerization path on bovine rhodopsin with a stiff opsin moiety (only cavity residues are relaxed during the calculation), this discrepancy suggests that the contribution of the TS_DIR_ to the thermal activation path is less significant in canonical cone pigments, which have a more fluctuating opsin moiety than rhodopsin. The number of molecular vibrational modes contributing thermal energy to pigment activation, *m*, is estimated to be 103. This is consistent with the previous study which reported that *m* is up to 141 (27). However, this estimated value (*i.e.*, *m* = 103) is larger than the value estimated in Luo *et al*. (*i.e.*, *m* = 45) (31). The vibrational modes arise from the interatomic forces and the bond vibrations within the protein structure, which are affected by environmental conditions of the protein. It is important to note that our studies evaluated the thermal activation rates of visual pigments incorporated into detergent micelles, whereas the previous electrophysiological studies assessed the rates of the pigments embedded in the membranes of photoreceptor cells. We speculate that these different conditions of the proteins (*i.e.*, membranes versus detergent micelles) can explain the discrepancy of the *m* values (*i.e.*, 45 *versus* 103). To confirm this speculation, it will be necessary to investigate the thermal activation rates of visual pigments reconstituted in membrane environments such as nanodiscs and styrene-maleic acid lipid particles in future work.

In conclusion, we quantitatively determined the contribution of the two factors, the λ_max_ and preexponential factor *A*, to the low thermal activation rates of rhodopsin and noncanonical cone pigments based on the Hinshelwood distribution-based model. The low thermal activation rate of nocturnal gecko UV can be attributed to both the blue-shifted λ_max_ and the low *A* value, whereas the low rates of rhodopsin, frog blue and nocturnal gecko green can primarily be ascribed to the low *A* value. This provides an evolutionary model in which noncanonical cone pigments convergently acquired a distribution pattern (expression in the rods) and molecular property (low thermal activation rate) similar to those of rhodopsin to participate in scotopic vision. In addition, among the visual pigments working in the rods, rhodopsin exhibits the lowest *A* value, resulting in the lowest thermal activation rate, due to the specific acquisition of Glu122 and Ile189 on the surface of the RBP. This well-conserved mechanism, quite low structural fluctuation, in rhodopsin would contribute to the highly sensitive scotopic vision commonly utilized in vertebrates.

## Experimental procedures

### Heterologous expression and purification of the visual pigments

The complementary DNA of bovine rhodopsin (K00506) was inserted into the mammalian expression vector, pUSRα. The complementary DNAs of other cone visual pigments, namely *Xenopus* violet (NM_001126076), chicken violet (M92039), and zebrafish UV (AF109373), were tagged by the epitope sequence of the anti-bovine rhodopsin monoclonal antibody Rho1D4 at the C terminus and inserted into the mammalian expression vector pMT4 or pcDNA3.1 (43). HEK293S cells were kindly provided by Prof. Jeremy Nathans (Johns Hopkins University School of Medicine) and were authenticated by short tandem repeat profiling. Expression of the visual pigments in HEK293S cells and sample preparation of the visual pigments were performed as previously described (14, 25, 26). The cell membranes expressing the visual pigments were divided into two aliquots. One was regenerated by 11-*cis* retinal and 7-membered-ring 11-*cis* retinal (7mr) (14, 44), and the other was regenerated by only 7mr. After regeneration, they were solubilized using Buffer A (50 mM Hepes, 140 mM NaCl, pH 6.5) containing 1% dodecyl maltoside (DDM) and purified using Rho1D4-conjugated agarose. The purified visual pigments were eluted with 0.02% DDM in Buffer A containing the synthetic C-terminal peptide of bovine rhodopsin. All experiments after reconstitution of the visual pigments with 11-*cis* retinal were performed in complete darkness using an infrared night vision device. We referred to the purified samples regenerated by both 11-*cis* retinal and 7mr, or only 7mr as “pigment name-n” or “pigment name-7mr”, respectively. We confirmed that the concentrations of the visual pigments contained in the two samples were similar by Western blotting analysis as previously described (14, 25, 26). The samples of the visual pigments for the spectroscopic measurement were regenerated with 11-*cis* retinal and purified as described above.

### Measurement of *v*_dark_, *v*_light_, *k*_d_ and estimation of *k*_th_

The *v*_dark_ was measured by [^35^S]GTPγS binding assay in the complete darkness using an infrared night vision device as previously described (14, 25, 26). After a 10 min incubation at 20 °C, the GDP/GTPγS exchange reaction was started by adding Gt solution purified from bovine retina. The assay mixture consisted of 300 nM pigments (1875 nM for zebrafish UV to increase signal-to-noise ratio), 1 μM Gt, 5 μM GTPγS, 25 nM [^35^S]GTPγS, 0.015% DDM, 50 mM Hepes (pH 6.5), 140 mM NaCl, 5.8 mM MgCl_2_, and 1 mM DTT. After incubation for 0 (immediately after mixture), 10, 20, and 30 min at 20 °C, 5 μl of the assay mixture was added into the stop solution [200 μl: 5 μM GTPγS in Buffer B (20 mM tris(hydroxymethyl)aminomethane (pH 7.4), 100 mM NaCl, 25 mM MgCl_2_)] to terminate the GDP/[^35^S]GTPγS exchange reaction. Then, the sample in the stop solution was immediately filtered through a nitrocellulose membrane to trap [^35^S]GTPγS bound to G proteins, and after that, the membrane was immediately washed 3 times with Buffer B to remove the nonspecific bound [^35^S]GTPγS. The amount of [^35^S]GTPγS was quantitated by assaying the membrane filter with a liquid scintillation counter. Experimental data were fitted by a single exponential function and *v*_dark_ was estimated by the difference between the initial rates between two samples (“pigment name-n” and “pigment name-7mr”).

The *v*_light_ was measured by a fluorescence assay as previously described (45, 46). The assay mixture consisted of 20 nM pigments (125 nM for zebrafish UV to increase signal-to-noise ratio), 0 or 1 μM Gt, 5 μM GTPγS, 0.015% DDM, 50 mM Hepes (pH 6.5), 140 mM NaCl, 5.8 mM MgCl_2_, and 1 mM DTT. Fluorescence signals were monitored at 20 °C using a laboratory-constructed photon counting system (45, 46). The excitation beam (300 nm) was generated using a Jasco J-600 spectropolarimeter, while fluorescence was detected using a photon counting head (H7360-01, Hamamatsu Photonics) connected to the controller unit (C8855, Hamamatsu Photonics). Fluorescence at greater than 310 nm was collected using an optical filter (U-360) in front of the photon counting head. The counting duration was 100 ms. The pigments were irradiated with a flashlight generated by a combination of a short arc xenon flash lamp (SA-200F, Nissin Electronic) and cutoff filters (Y52 filter for bovine rhodopsin, L42 for chicken violet and *Xenopus* violet, and without a filter for zebrafish UV). Experimental data were fitted by a single exponential function to estimate *k*_r_*′*, *ΔF*_1*,*_ and *ΔF*_2_, representing the initial slope of the fluorescence increase in the presence of Gt, the total fluorescence increase in the presence of Gt, and the total fluorescence increase in the absence of Gt. *v*_light_ was estimated as previously described (45, 47) using the following equation:

(Eq. 7) $$v_{\mathrm{light}}={k_{r}}^{\prime }\;\left(\Delta F_{1}-\Delta F_{2}\right)$$

The *k*_d_ was measured by a fluorescence assay as previously described (47, 48). The assay mixture consisted of 20 nM pigments (60 nM for chicken violet and *Xenopus* violet and 375 nM for zebrafish UV to increase signal-to-noise ratio), 5 μM GTPγS, 0.015% DDM, 50 mM Hepes (pH 6.5), 140 mM NaCl, 5.8 mM MgCl_2_, and 1 mM DTT. Fluorescence signals of bovine rhodopsin were monitored at 20 °C using a conventional fluorophotometer (Shimadzu RF-5300PC). Excitation and emission wavelengths were set at 295 and 340 nm, respectively. The pigments were irradiated with yellow light generated by a combination of a 1 kW halogen lamp (Master HILUX-HR; Rikagaku) and a Y52 cutoff filter. Fluorescence signals of chicken violet, *Xenopus* violet, and zebrafish UV were monitored at 20 °C using the laboratory-constructed photon counting system described above where excitation wavelength was set at 295 nm and fluorescence greater than 310 nm was collected using an optical filter (U-360). The pigments were irradiated with a flashlight as described above. Experimental data were fitted by a single exponential function to estimate *k*_d_. As described in our previous reports, the thermal isomerization rates (*k*_th_) were calculated from Equation 4 by using three experimentally determined values (*v*_dark_, *v*_light,_ and *k*_d_).

### Western blotting

Extracts from visual pigment-transfected HEK293S cells were subjected to SDS-PAGE, transferred onto a polyvinylidene difluoride membrane, and probed with the monoclonal antibody Rho1D4. Immunoreactive proteins were detected using enhanced chemiluminescence (GE HealthCare) and visualized using a luminescent image analyzer (LAS 4000mini, GE HealthCare) as previously described (14, 25, 26).

### Spectroscopic measurements

Absorption spectra of the samples were recorded with a UV–visible spectrophotometer (Shimadzu UV-2450, UV-2400). Samples were kept at 0 °C using a cell holder equipped with a temperature-controlled circulating water bath.

### Data analysis

Data analysis was conducted using Igor Pro 8 software (version 8.0.4, WaveMetrics https://www.wavemetrics.com/). The thermal activation rates of canonical cone pigments were fitted by the Hinshelwood model (Equation 1) assuming different numbers of molecular vibrational modes (1 ≤ *m* ≤ 141) with *A* as a variable and *T* and *E*_a_ fixed at 293.15 K and 0.84 × *h*c/λ_max_, respectively. For each fitting model, AIC was calculated as follows:

(Eq. 8) $$\text{AIC}=n\left(\ln\left(2\pi \sigma^{2}\right)+1\right)+2\left(p+1\right)$$

where *n* is the number of data points for the curve fitting, *σ*^2^ is the residual sum of squares, and *p* is the number of free parameters. The model with the lowest AIC (22.9) was then adopted. In this model, *m* and *A* were estimated to be 103 and 9.23 × 10^−6^ s^−1^, respectively. The same data were also fitted by the Arrhenius model (Equation 6) where *E*_a_ is proportional to the reciprocal of the pigment’s λ_max_ (*E*_a_ = a/λ_max_ + b kcal/mol). The fitting analysis was performed with both a and b as variables and *T* and a/500 + b fixed at 293.15 K and 22 kcal/mol, respectively (7). Following this fitting procedure, the values of *E*_a_*, A* and AIC were estimated to be 1526/λ_max_ + 18.95 kcal/mol, 2.76 × 10^11^ sec^−1^, and 17.6, respectively.

## Data availability

All data are available in the main text or the supporting information.

## Supporting information

This article contains supporting information (14, 25, 26, 49).

## Conflict of interest

The authors declare that they have no conflicts of interest with the contents of this article.
