# Supplementary material for: Convergent mechanism underlying the acquisition of vertebrate scotopic vision
Source: J Biol Chem. 2024 Mar 16;300(4):107175. doi: 10.1016/j.jbc.2024.107175 (PMC11007431; doi:10.1016/j.jbc.2024.107175)
Supplement: Supporting Figures S1–S6 and Table S1 [file mmc1.docx]

Supporting information for

Convergent mechanism underlying the acquisition of vertebrate scotopic vision

Keiichi Kojima^1,2^, Masataka Yanagawa^3,4^, Yasushi Imamoto^1^, Yumiko Yamano^5^, Akimori Wada^6^, Yoshinori Shichida^1,7^, Takahiro Yamashita^1^*

^1^ Department of Biophysics, Graduate School of Science, Kyoto University, Kyoto 606-8502, Japan.

^2^ Faculty of Medicine, Dentistry and Pharmaceutical Sciences, Okayama University, Okayama 700-8530, Japan.

^3^ Cellular Informatics Laboratory, RIKEN Cluster for Pioneering Research, 2-1 Hirosawa, Wako 351-0198, Japan.

^4^ Molecular and Cellular Biochemistry, Graduate School of Pharmaceutical Sciences, Tohoku University, Sendai, Miyagi, 980-8578, Japan.

^5^ Comprehensive Education and Research Center, Kobe Pharmaceutical University, Kobe 658-8558, Japan.

^6^ Laboratory of Organic Chemistry for Life Science, Kobe Pharmaceutical University, Kobe 658-8558, Japan.

^7^ Research Organization for Science and Technology, Ritsumeikan University, Kusatsu, Shiga 525-8577, Japan

Corresponding Author: Takahiro Yamashita, Department of Biophysics, Graduate School of Science, Kyoto University, Kyoto 606-8502, Japan. yamashita.takahiro.4z@kyoto-u.ac.jp


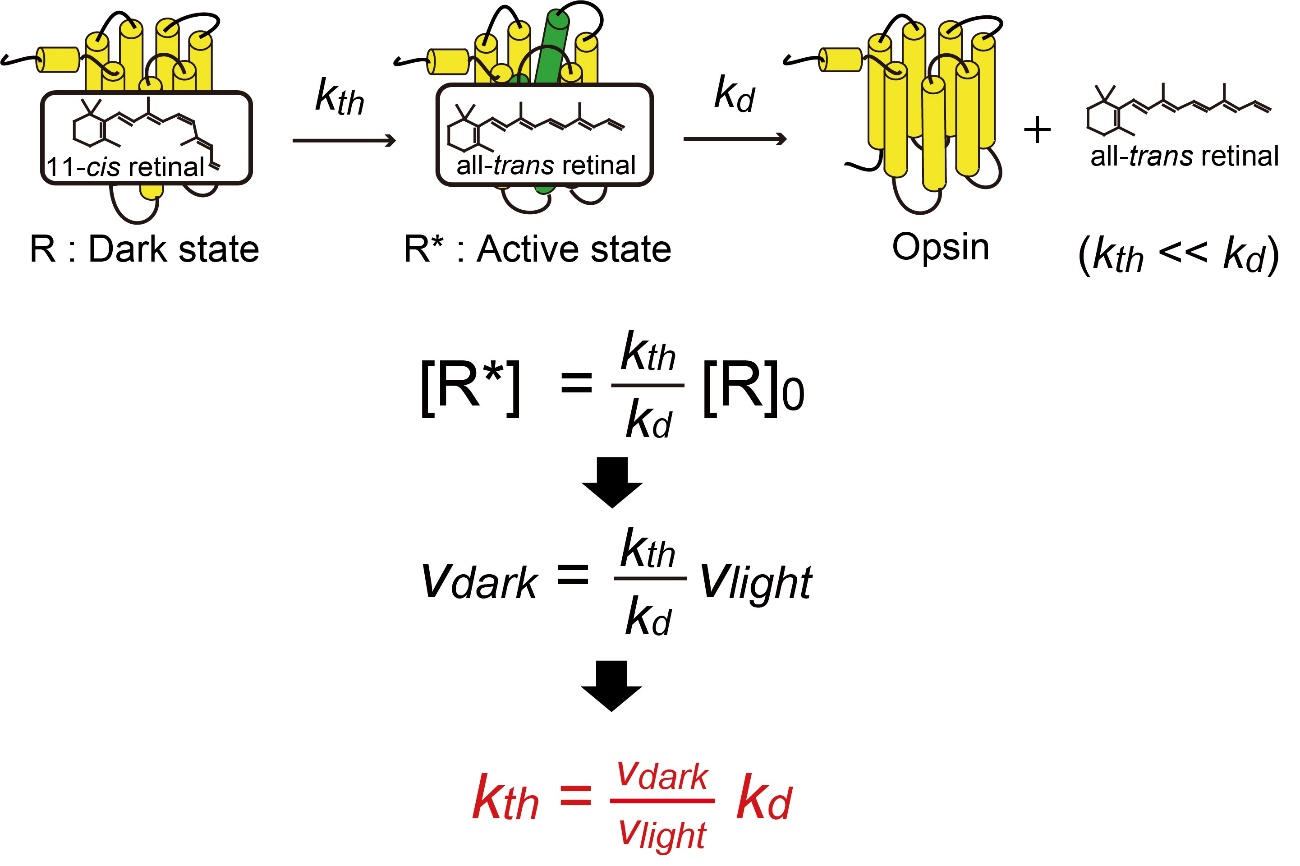
**Supplementary Figures**

**Supplementary Figure 1. Calculation of thermal activation rates of visual pigments.** Two-step reaction scheme of thermal activation and deactivation of visual pigments. R and R* indicate visual pigments in the dark (inactive) and active states, respectively. *k*_th_ indicates a thermal activation rate constant of R. *k*_d_ indicates a thermal decay rate constant of R*. An opsin regenerated with normal 11-*cis* retinal spontaneously converts to R* by thermal isomerization of retinal chromophore in the dark. After the first reaction, R* is degraded into opsin and retinal. Given that the thermal activation of R occurs much more slowly than the decay of R* (*k*_th_ << *k*_d_), a steady-state approximation can be applied to the concentration of R* and the equations in the figure are obtained.


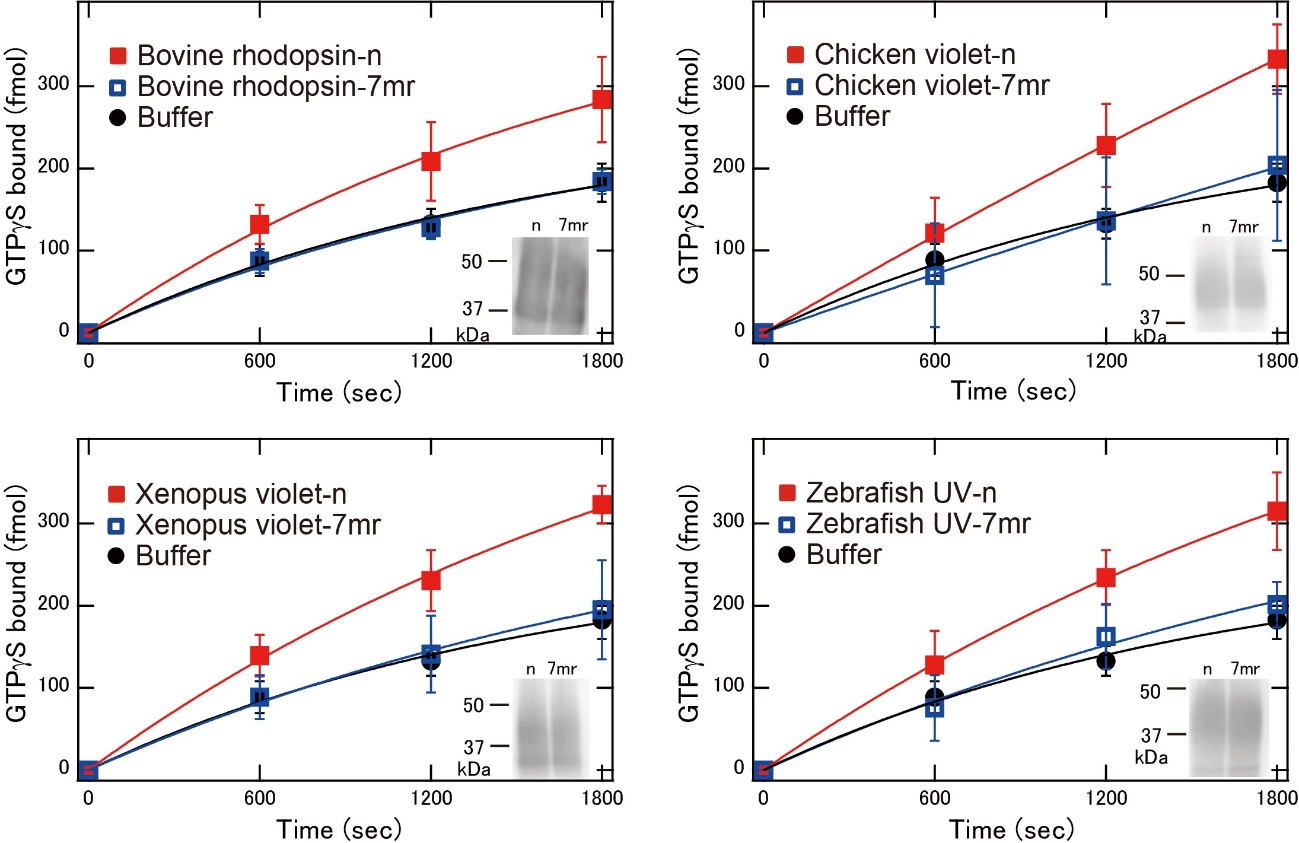


**Supplementary Figure 2. Measurements of *v*_dark_ by a [^35^S]GTPγS binding assay.** Data from bovine rhodopsin-n, chicken violet-n, Xenopus violet-n, salamander UV-n, zebrafish UV-n, and garter snake UV-n are indicated by filled red squares. Data from bovine rhodopsin-7mr, chicken violet-7mr, Xenopus violet-7mr, salamander UV-7mr, zebrafish UV-7mr, and garter snake UV-7mr are indicated by open blue squares. The buffer control is indicated by filled black circles. *n* = 3 (zebrafish UV, tiger salamander UV, garter snake UV and green anole green), 4 (green anole UV, chicken violet, zebrafish blue, xenopus violet, newt blue, chicken green and mouse green), and 5 (bovine rhodopsin). Error bars represent the S.D. The western blotting data were cropped and are shown in the inset (left lane: purified samples regenerated by both 11-*cis* retinal and 7mr, right lane: purified samples regenerated by only 7mr).


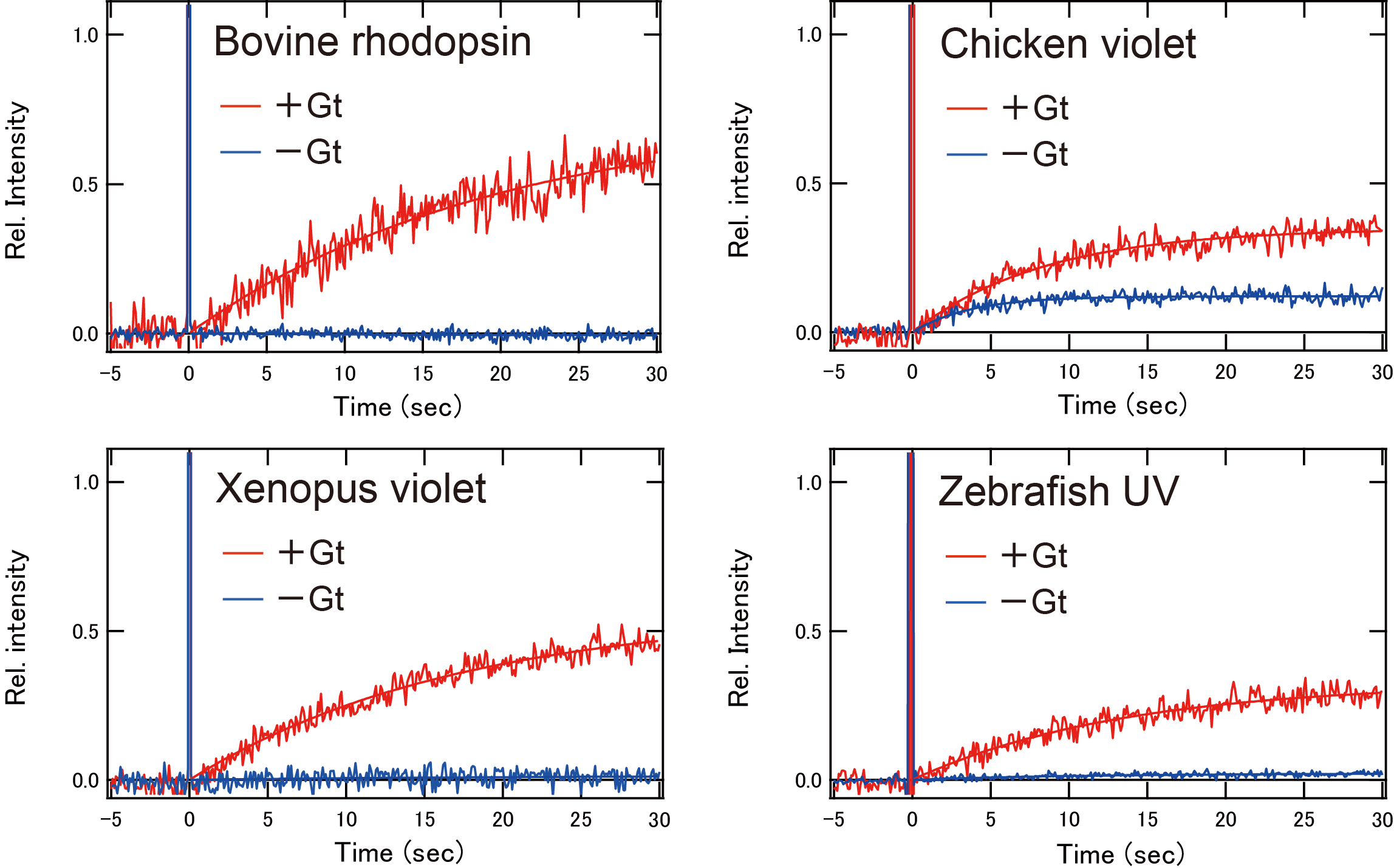


**Supplementary Figure 3. Measurements of *v*_light_ by monitoring the change of intrinsic tryptophan fluorescence.** The change of intrinsic tryptophan fluorescence after light irradiation with or without Gt is indicated by the red or blue line, respectively. Intensities were normalized by the full fluorescence increase in the presence of aluminum fluoride.


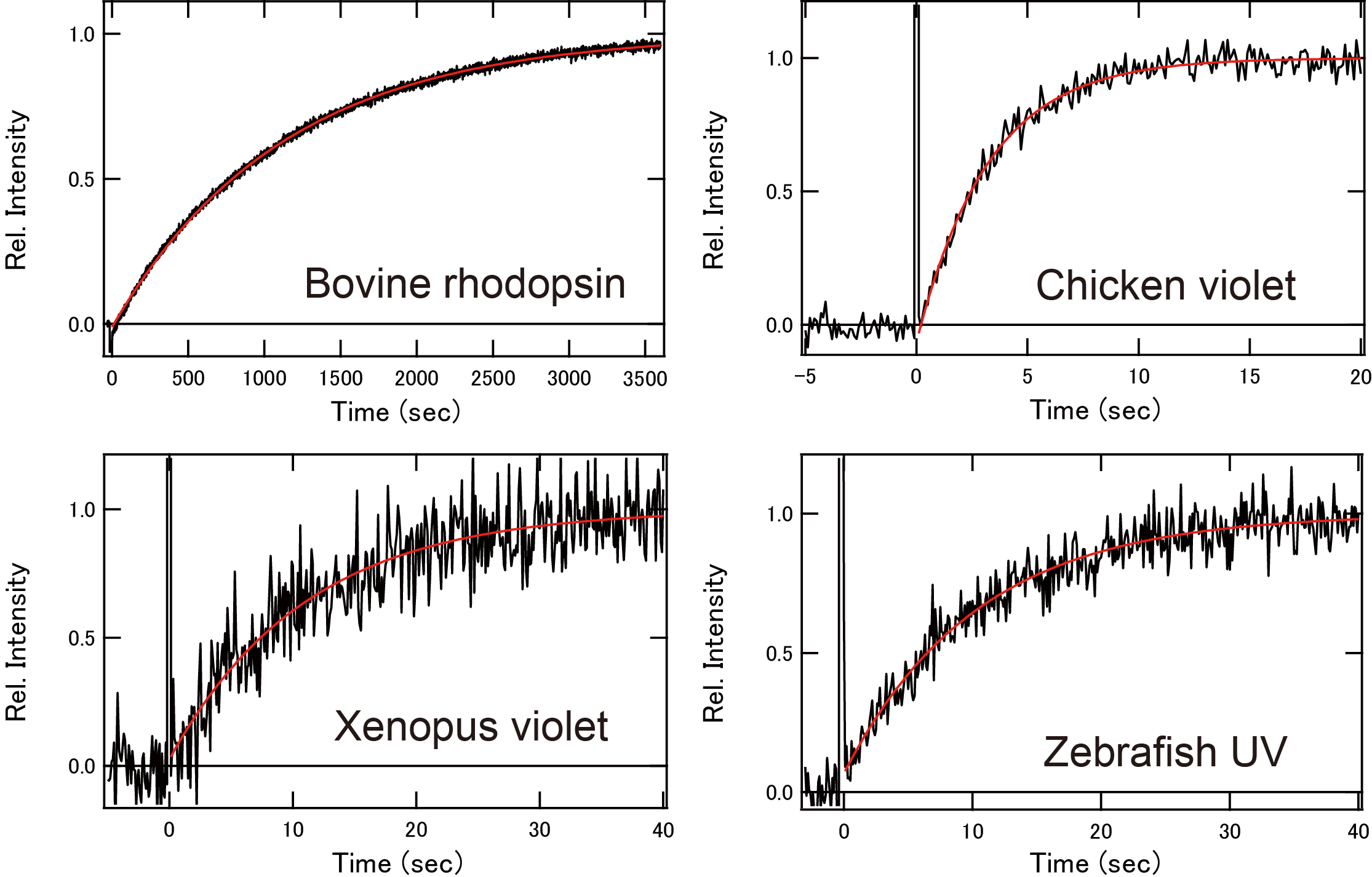


**Supplementary Figure 4. Measurements of *k*_d_ by monitoring the change of intrinsic tryptophan fluorescence.** The change of intrinsic tryptophan fluorescence after light irradiation without Gt is indicated by the black line.


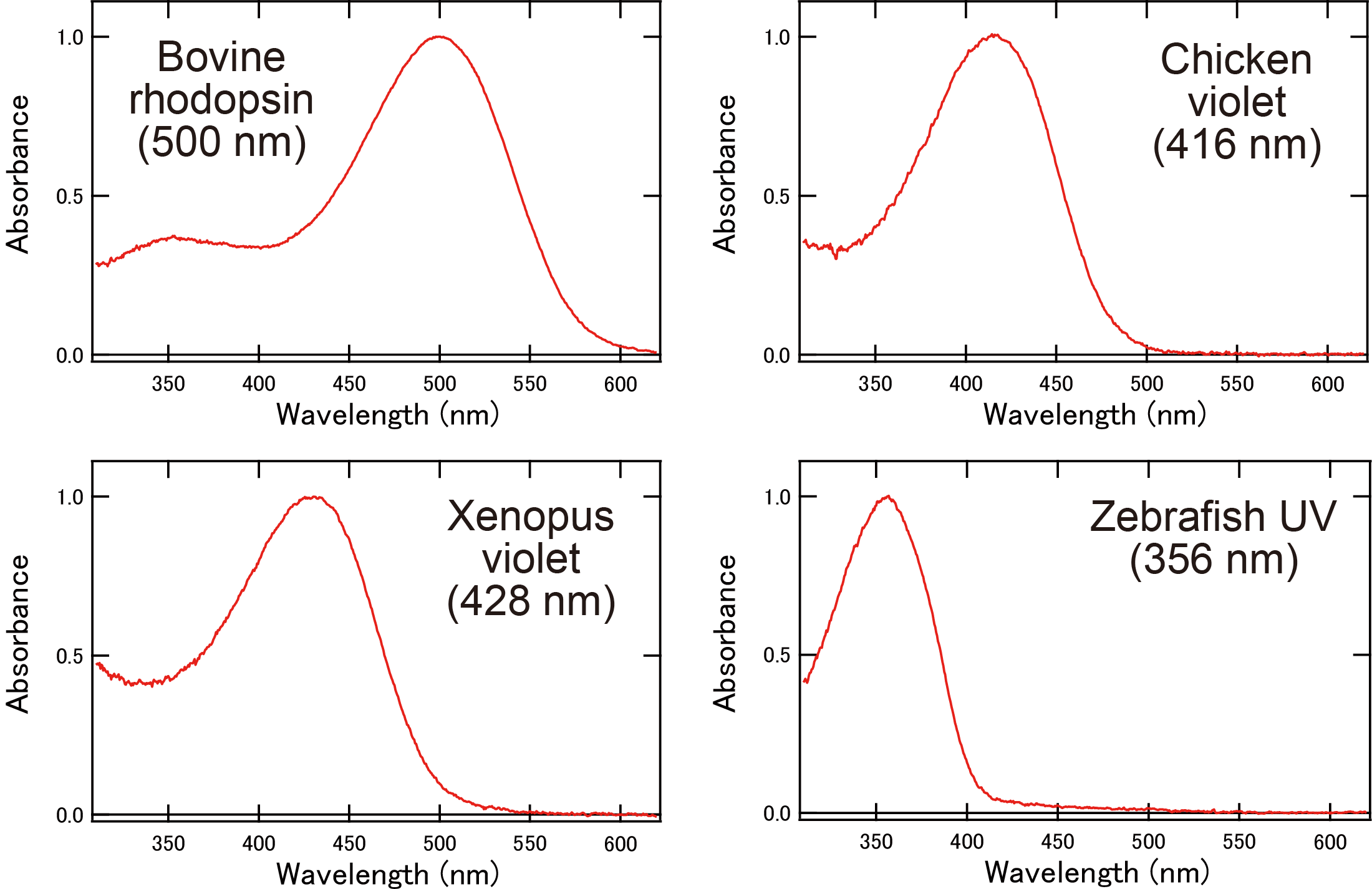


**Supplementary Figure 5. Absorption spectra of visual pigments after reconstitution with 11-*cis* retinal.** Spectra were normalized at peak absorbance.


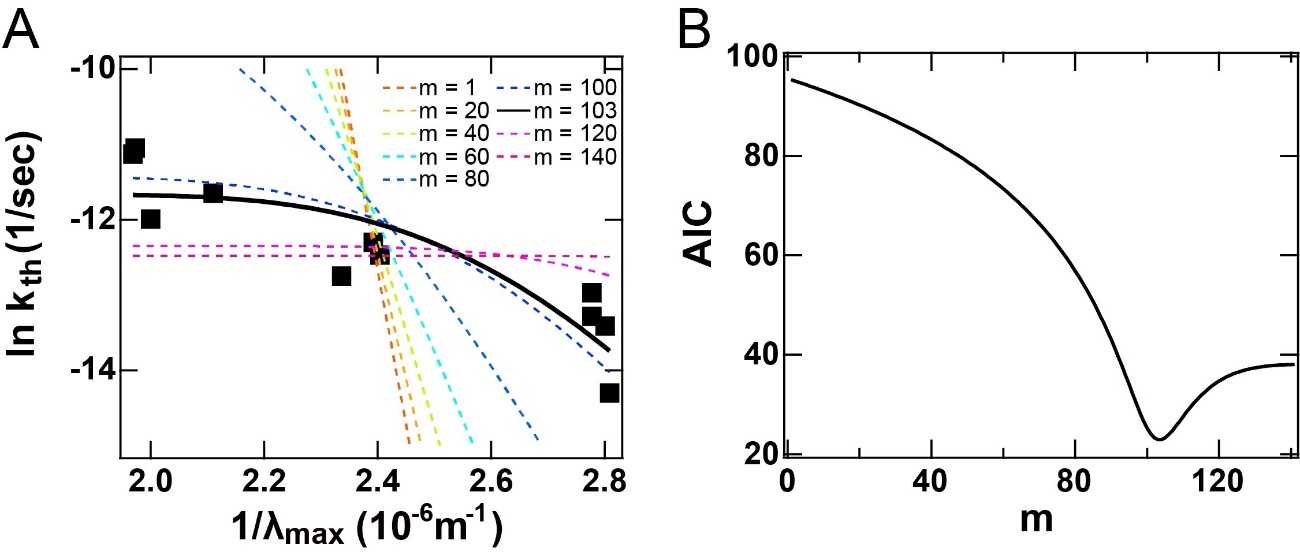


**Supplementary Figure 6. Fitting of the thermal activation rates of canonical cone pigments with the Hinshelwood distribution-based model.**

(A) Fitting curve of the Hinshelwood distribution-based model with *m* = 1, 20, 40, 60, 80, 100, 103, 120 and 140. Black squares show the thermal activation rates of canonical cone pigments (i.e., mouse green, chicken green, green anole green, newt blue, zebrafish blue, chicken violet, Xenopus violet, green anole UV, garter snake UV, tiger salamander UV and zebrafish UV). (B) AIC values of the Hinshelwood distribution-based model.


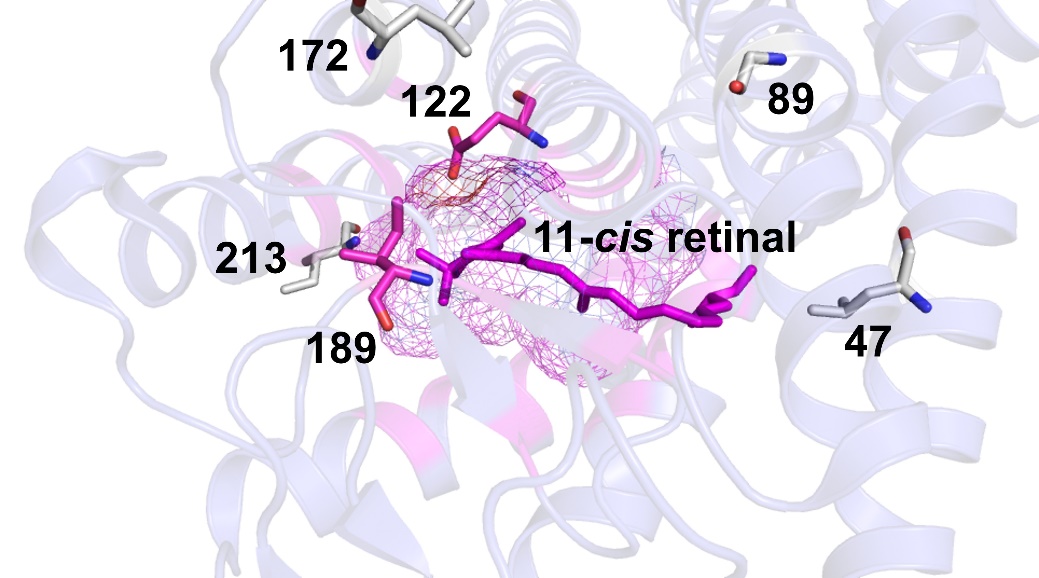


**Supplementary Figure 7. Key amino acid residues for the low thermal activation rates in rhodopsins and noncanonical cone pigments.** Glu122 and Ile189 are located on the surface of the RBP. This figure was constructed based on the crystal structures of the dark state of bovine rhodopsin (1U19 (1)). The cavities around the RBP are shown in the magenta mesh.

**Table S1**

Thermal activation rate constants based on the Arrhenius model and absorption maxima (λ_max_) of visual pigments.

| Group | Visual pigment | λ_max_ (nm) | exp(*-E_a_*/RT) ^‡^ | *A* ^§^ | *k*_th_ (1/sec)* |  |
| --- | --- | --- | --- | --- | --- | --- |
|  |  |  |  |  |  |  |
| Rhodopsin (RH1) | Bovine rhodopsin | 500 | 3.86×10^-17^ | 6.40×10^8^ | 2.47×10^-8^ |  |
|  | Mouse rhodopsin | 500 | 3.86×10^-17^ | 5.31×10^8^ | 2.05×10^-8^ |  |
|  | Xenopus rhodopsin | 500 | 3.86×10^-17^ | 4.12×10^8^ | 1.59×10^-8^ |  |
| L (LWS/MWS) | Mouse green | 508 | 4.19×10^-17^ | 3.48×10^11^ | 1.46×10^-5^ |  |
| M2 (RH2) | Chicken green | 507 | 4.15×10^-17^ | 3.86×10^11^ | 1.60×10^-5^ |  |
|  | Green anole green | 500 | 3.86×10^-17^ | 1.61×10^11^ | 6.22×10^-6^ |  |
| M1 (SWS2) | Newt blue | 474 | 2.89×10^-17^ | 3.01×10^11^ | 8.71×10^-6^ |  |
|  | Zebrafish blue | 418 | 1.38×10^-17^ | 3.39×10^11^ | 4.67×10^-6^ |  |
| S (SWS1) | Xenopus violet | 428 | 1.60×10^-17^ | 1.82×10^11^ | 2.91×10^-6^ |  |
|  | Chicken violet | 416 | 1.34×10^-17^ | 2.85×10^11^ | 3.82×10^-6^ |  |
|  | Green anole UV | 360 | 5.02×10^-18^ | 3.38×10^11^ | 2.33×10^-6^ |  |
|  | Garter snake UV | 360 | 5.02×10^-18^ | 4.64×10^11^ | 1.70×10^-6^ |  |
|  | Tiger salamander UV | 357 | 4.72×10^-18^ | 3.17×10^11^ | 1.50×10^-6^ |  |
|  | Zebrafish UV | 356 | 4.63×10^-18^ | 1.33×10^11^ | 6.15×10^-7^ |  |
| M1 (SWS2) | Xenopus blue | 432 | 1.69×10^-17^ | 3.06×10^9^ | 5.17×10^-8^ |  |
|  | American bullfrog blue | 431 | 1.67×10^-17^ | 6.30×10^9^ | 1.05×10^-7^ |  |
|  | Mantelline frog blue | 432 | 1.69×10^-17^ | 4.01×10^9^ | 6.78×10^-8^ |  |
| M2 (RH2) | Tokay gecko green | 467 | 2.66×10^-17^ | 9.39×10^9^ | 2.50×10^-7^ |  |
| S (SWS1) | Tokay gecko UV | 360 | 5.02×10^-18^ | 3.28×10^10^ | 1.65×10^-7^ |  |

*The thermal activation rates (*k*_th_) of visual pigments are the experimental data estimated using the biochemical methods in this study and our previous studies (2-4).

^‡^The exp(-*E_a_*/RT) values were calculated using Eq. 6.

^§^The *A* values were calculated based on their thermal activation rates and exp(-*E_a_*/RT) values.

**References**

1. Okada, T., Sugihara, M., Bondar, A. N., Elstner, M., Entel, P., and Buss, V. (2004) The retinal conformation and its environment in rhodopsin in light of a new 2.2 Å crystal structure. *J. Mol. Biol.* **342**, 571-583

2. Yanagawa, M., Kojima, K., Yamashita, T., Imamoto, Y., Matsuyama, T., Nakanishi, K.*, et al.* (2015) Origin of the low thermal isomerization rate of rhodopsin chromophore. *Sci. Rep.* **5**, 11081

3. Kojima, K., Matsutani, Y., Yamashita, T., Yanagawa, M., Imamoto, Y., Yamano, Y.*, et al.* (2017) Adaptation of cone pigments found in green rods for scotopic vision through a single amino acid mutation. *Proc. Natl. Acad. Sci. USA* **114**, 5437-5442

4. Kojima, K., Matsutani, Y., Yanagawa, M., Imamoto, Y., Yamano, Y., Wada, A.*, et al.* (2021) Evolutionary adaptation of visual pigments in geckos for their photic environment. *Sci. Adv.* **7**, eabj1316
